# Aminoacyl-tRNA Synthetases as Valuable Targets for Antimicrobial Drug Discovery

**DOI:** 10.3390/ijms22041750

**Published:** 2021-02-10

**Authors:** Luping Pang, Stephen D. Weeks, Arthur Van Aerschot

**Affiliations:** 1 KU Leuven, Rega Institute for Medical Research, Medicinal Chemistry, Herestraat 49–box 1041, 3000 Leuven, Belgium; Luping.Pang@kuleuven.be; 2 KU Leuven, Biocrystallography, Department of Pharmaceutical and Pharmacological Sciences, Herestraat 49–box 822, 3000 Leuven, Belgium; 3 Pledge Therapeutics, Gaston Geenslaan 1, 3001 Leuven, Belgium; sweeks@pledge-tx.com

**Keywords:** antimicrobial resistance, aminoacyl-tRNA synthetases, anti-infective targets, structure-based drug design, intermediate analogs, albomycin, microcin C, antibiotic, antibacterial, antifungal

## Abstract

Aminoacyl-tRNA synthetases (aaRSs) catalyze the esterification of tRNA with a cognate amino acid and are essential enzymes in all three kingdoms of life. Due to their important role in the translation of the genetic code, aaRSs have been recognized as suitable targets for the development of small molecule anti-infectives. In this review, following a concise discussion of aaRS catalytic and proof-reading activities, the various inhibitory mechanisms of reported natural and synthetic aaRS inhibitors are discussed. Using the expanding repository of ligand-bound X-ray crystal structures, we classified these compounds based on their binding sites, focusing on their ability to compete with the association of one, or more of the canonical aaRS substrates. In parallel, we examined the determinants of species-selectivity and discuss potential resistance mechanisms of some of the inhibitor classes. Combined, this structural perspective highlights the opportunities for further exploration of the aaRS enzyme family as antimicrobial targets.

## 1. Introduction

The discovery and development of antimicrobial compounds to treat infectious diseases has changed the therapeutic paradigm and revolutionized modern medicine. It is undoubtedly true that antimicrobials have been recognized as one of the most successful medical interventions due to their indispensable role in complex medical approaches such as organ transplantation, cancer treatment, cutting edge surgical preparation, chronic diseases, and many others [1]. Their application has dramatically reduced human morbidity and mortality caused by infectious diseases, and simultaneously extended life span in the last century [2]. Although extremely efficacious, the rapid emergence and spread of resistant bacteria, occurring at a global scale, is endangering the use of antibiotics and is now recognized as one of the major global public health threats of the 21st century [3]. This predicament has been attributed to the overuse and misuse of these medicines as well as to a lack of new drug development by the pharmaceutical industry over the last few decades. Medical analysts and the World Health Organization (WHO) are now warning of the potential danger of re-entering the dark times of the pre-antibiotic era.

Antimicrobial resistance (AMR) has been detected for almost all antibiotic classes such as penicillins, tetracyclines, cephalosporins, quinolones, aminoglycosides, and macrolides. For some, resistance was observed even before their application in the clinic [4]. Herein, resistance to a particular class of antimicrobials can often be attributed to multiple biochemical pathways. Excellent reviews of the various mechanisms of AMR have been published elsewhere and so will not be discussed here [5,6,7,8,9]. Infections caused by AMR or multidrug resistance (MDR) are an enormous health and economic burden not only to the public healthcare system, but also to the patients and their families. A recent review commissioned by the UK government estimated that global deaths resulting from AMR could increase from approximately 700,000 annually to close to 10 million deaths by 2050, and lead to a cumulative USD 100 trillion loss to the global economy if these infections are left untreated [10]. Moreover, according to a recent WHO report, several bacterial strains including carbapenem-resistant *Acinetobacter baumannii* and *Pseudomonas aeruginosa*, and carbapenem-resistant, extended spectrum β-lactamase (ESBL)-producing *Enterobacteriaceae* have been recognized as extremely dangerous pathogens that demonstrate resistance to the vast majority of known antibiotics [11]. These infectious agents already present significant medical challenges including longer hospital stays, treatment failures, increased medical costs, lengthier recuperations, and side-effects resulting from off-target associated toxicity of last-resort drugs. Such infections, therefore, are in urgent need of effective new remedies.

The vast majority of clinically used antibacterial compounds target essential bacterial functions or growth processes. They can disrupt or interfere with bacterial cell wall synthesis, cell membrane permeability, or inhibit crucial biochemical enzymes or apparatuses involved in the synthesis of DNA, RNA, or protein. Aminoacyl-tRNA synthetases (aaRSs) are a family of essential and universal ‘house-keeping’ enzymes responsible for catalyzing the esterification of amino acids to their cognate tRNAs. The produced charged tRNAs are necessary for decoding the genetic information during protein synthesis [12,13,14]. In addition to this canonical function, recent studies have shown that aaRSs are also involved in numerous noncanonical activities including transcription regulation, signal transduction, angiogenesis, and other aspects of cellular homeostasis [15,16]. This emerging dimension of aaRS biology has identified important roles of both cytoplasmic and mitochondrial aaRSs in diverse human diseases [15,17]. Crucially, as key components of protein translation, aaRSs from pathogenic microorganisms are recognized as targets for the discovery of new antimicrobials.

In this review, a brief description of the catalytic and proof-reading mechanisms of aaRSs is followed by an overview of the multiple strategies employed by various natural and synthetic aaRS inhibitors. We focus on how these different types of inhibitors bind and interact with the corresponding target aaRSs by examining their potential species-selective mechanisms as well as the occurrence of resistance. We will then discuss the ways in which the proposed strategies aim for increasing the whole cell activities, reducing host cell toxicities and how the emergence of resistance to these aaRS inhibitors can be reduced or circumvented.

To clarify terminology, an antibiotic originally referred to a natural product obtained from a microorganism that could inhibit the growth of or kill bacteria, while an antibacterial is defined as any substance effective against bacteria where its source can be natural, semi-synthetic, or synthetic in origin. Currently however, “antibiotic” is often used as a synonym for “antibacterial” [18] or the broader term antimicrobial, which encompasses all classes of anti-infectives. These terms will be used interchangeably throughout this review.

## 2. Catalytic Mechanism of Aminoacyl-tRNA Synthetases (aaRSs)

**Figure 1:**
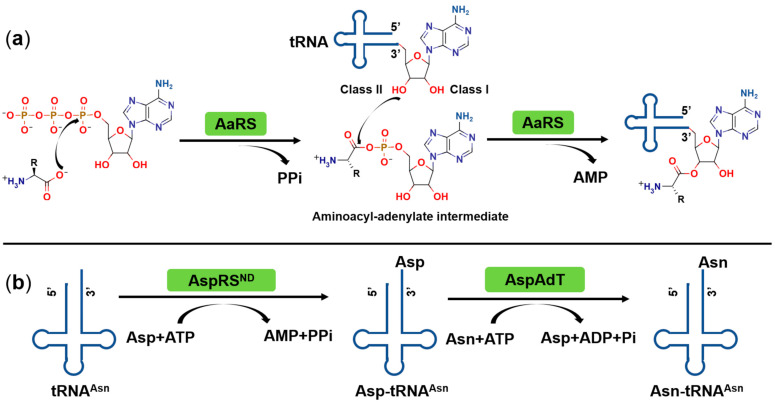
Catalytic mechanisms for the standard (**a**) and indirect (**b**) aminoacylation reaction.

AaRSs catalyze the highly specific aminoacylation reaction in a common two-step reaction [13,19] (Figure 1a). In the first step, the α-carboxylate oxygen of the amino acid attacks the α-phosphate of ATP requiring Mg^2+^ as a co-factor, forming an aminoacyl-adenylate (aa-AMP) intermediate and releasing the byproduct inorganic pyrophosphate (PPi). In the subsequent reaction, the activated amino acid is transferred to the 2′- or 3′-hydroxyl group of the ribose moiety of the 3′-terminal adenosine of the corresponding tRNA with the release of AMP (Figure 1a). The produced aminoacyl-tRNA (aa-tRNA) is used as a substrate by the ribosome for the de novo synthesis of proteins. Typically, these two steps can be considered independently. However, the arginyl-, glutaminyl-, glutamyl-tRNA synthetases (ArgRS, GlnRS, and GluRS, respectively) and some unusual archaeal lysyl-tRNA synthetases (LysRS) can only catalyze aa-AMP formation in the presence of cognate tRNA [20,21,22,23]. For these four enzymes, their cognate tRNAs play a dual role during catalysis, acting as an activator in the first reaction and as a substrate in the aminoacyl transfer process.

In most prokaryotes, tRNA aminoacylation is ensured by 20 different aaRSs, one for each proteinogenic amino acid [13,19]. However, asparaginyl-tRNA synthetase (AsnRS) and glutaminyl-tRNA synthetase (GlnRS), responsible for specific Asn-tRNA^Asn^ and Gln-tRNA^Gln^ formation, are missing in certain microorganisms, and in the latter case also in chloroplasts [24]. Their functions are substituted by a non-discriminating aspartyl-tRNA synthetase (AspRS^ND^) and glutamyl-tRNA synthetase (GluRS^ND^), respectively [24]. These enzymes catalyze the formation of mischarged Asp-tRNA^Asn^ and Glu-tRNA^Gln^, which are then converted to Asn-tRNA^Asn^ and Gln-tRNA^Gln^ by tRNA-dependent amidotransferases (AdT) [25,26,27] (Figure 1b).

Apart from the 20 canonical aaRSs, two unique aaRSs have been identified in archaea known as phosphoseryl-tRNA synthetase (SepRS) [28] and pyrrolysyl-tRNA synthetase (PylRS) [29] that expand the genetic code for noncanonical amino acids. In some methanogenic archaeal organisms, SepRS ligates phosphoserine onto tRNA^Cys^, which is then converted to Cys-tRNA^Cys^ by the Sep-tRNA:Cys-tRNA synthase [30]. This full aminoacylation reaction is conceptually similar to the aforementioned indirect catalysis of Asn-tRNA^Asn^ and Gln-tRNA^Gln^. In the case of pyrrolysine (Pyl), it is inserted in the protein sequence corresponding to an inframe UAG stop codon by tRNA^Pyl^, which itself is produced by PylRS. Interestingly, PylRS and tRNA^Pyl^ function as an orthogonal pair excluding any interactions with other aaRSs and tRNAs. Based on this finding, an enzyme engineering technique is widely used to generate PylRS variants aiming for incorporation of noncanonical amino acids into recombinant proteins [31,32]. In addition, the technique has recently been further improved and multiple chimeric tRNA synthetase/chimeric tRNA pairs were designed, allowing orthogonal and highly efficient incorporation of a large variety of aromatic and fluorescent amino acids into a biological produced product [33].

Compared to prokaryotes, humans encode 36 distinct tRNA synthetases: 16 cytoplasmic (including the bifunctional glutamyl-prolyl tRNA synthetase (EPRS) in charge of the aminoacylation of Glu and Pro), 17 mitochondrial, and three dual-localized aaRSs (GlnRS, GlyRS, and LysRS) present in both the cytoplasm and mitochondria [34]. In light of the primary sequence and the corresponding structure, cytoplasmic and mitochondrial aaRSs are considered to have archaeal and eubacterial origins, respectively. This likely reflects the endosymbiotic origin of eukaryotes, where a eubacterial cell was engulfed by archaea [35]. Reflecting these origins, the catalytic pathway of the human enzymes is identical to that found in prokaryotes (Figure 1).

## 3. Classification of aaRSs

**Figure 2:**
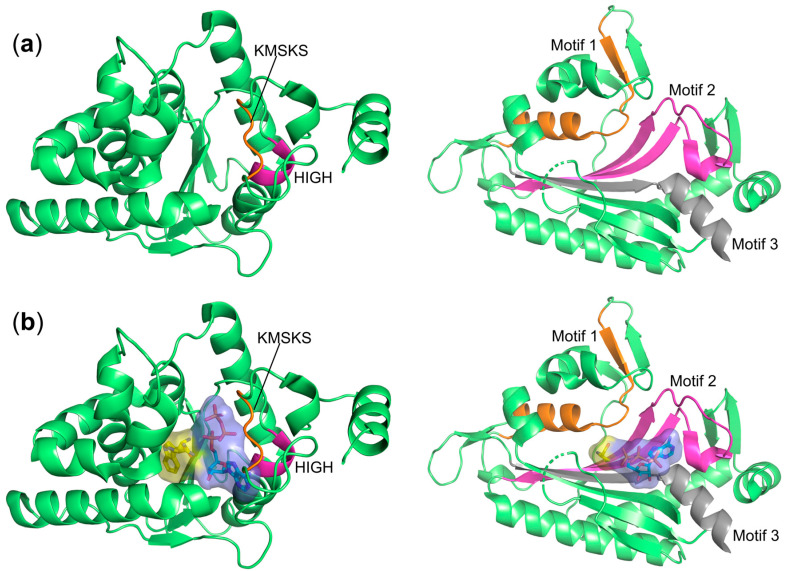
The architectures of the catalytic sites of class I and II aaRSs. (**a**) The catalytic domains and signature sequence motifs in class I (left) and class II (right) aaRSs. (**b**) The binding pockets of ATP and amino acid in class I (left) and class II (right) aaRSs. The crystal structure of *B. stearothermophilus* TrpRS (Bs-TrpRS) in complex with ATP and tryptophanamide (PDB entry: 1MAU) represents class I aaRSs while the structure of *Enterococcus faecalis* ProRS (Ef-ProRS) in complex with ATP and prolinol (PDB entry: 2J3M) as the representative for class II aaRSs. For clarity, we only show the catalytic domains corresponding to residues 1–221 for Bs-TrpRS and residues 19–214 and 405–456 for Ef-ProRS. Class I conserved HIGH and KMSKS sequence motifs are colored in magenta and orange, respectively. Motifs 1, 2, and 3 of the class II aaRSs are shown in orange, magenta, and grey, respectively. The binding cavities of ATP (slate) and amino acid (yellow) are shown as semi-transparent surface representations with ligands shown as sticks.

Due to their common biochemical activity, aaRS members were originally hypothesized to have evolved from a single common ancestor. This hypothesis was originally supported by crystal structures of aaRSs determined prior to 1989 including *Bacillus stearothermophilus* TyrRS [36], *E. coli* MetRS [37], and GlnRS [38], which showed significant structural homology between each other. Later, studies showed that two structurally distinct classes exist, and that the above representatives all coincidently belonged to the so-called class I group (Table 1). The catalytic domains of all class I aaRSs adopt a Rossmann fold (RF, also known as dinucleotide-binding fold) and the domain features a five-stranded parallel β-sheet connected by α-helices (Figure 2a,b). Class I enzymes are usually monomeric, possessing two conserved HIGH (His-Ile-Gly-His) and KMSKS (Lys-Met-Ser-Lys-Ser) signature sequences that are involved in ATP binding [14,39] (Figure 2b). At the primary sequence level, these two motifs cap the two ends of the catalytic domain. The HIGH sequence is located at the amino terminus being present in the first α-helix of the RF, whereas KMSKS is typically found in a loop just after the fifth strand close to the carboxyl-terminal of the RF (Figure 2). The anticodon binding domain, responsible for the binding of the tRNA anticodon region, is typically located downstream of the catalytic domain [40].

Sequence analysis has shown that the class I signature motifs only apply to 10 members of the 20 standard aaRSs. Using a limited number of sequences, the group of D. Moras defined the remaining 10 aaRS as a separate class II group (Table 1 and Figure 2) that all share three cryptically conserved motifs [41]. This classification was further supported by X-ray crystal structures of *E. coli* SerRS [42] and the yeast AspRS·tRNA^Asp^ complex [43], which demonstrated a very different catalytic core architecture to that found in class I enzymes (Figure 2). The common catalytic domain identified in all class II aaRSs is composed of a six-stranded β-sheet flanked by a number of α-helices (Figure 2a) and most class II members are homodimers. The three conserved motifs are important for the biological assembly and aminoacylation activity of class II aaRSs [40]. Briefly, motif 1 is composed of a long α-helix connected with a β-strand terminated by a highly conserved proline residue and is involved in the dimer interface, while motifs 2 and 3 form the ATP and amino acid binding sites where ATP is maintained in a horseshoe conformation (Figure 2b).

In addition to the structural differences, the two-group classification also reflects distinct biochemical properties of the members. Typically, the class I enzymes approach the tRNA acceptor stem from the minor groove and catalyze aminoacylation directly to the 2′-hydroxyl group of the terminal adenosine A76 [14,39,44]. In contrast, all class II family members approach the acceptor stem of tRNA from the major groove and transfer the amino acid to the 3′-hydroxyl of the terminal adenosine, with the exception of PheRS, which aminoacylates the 2′-hydroxyl of the tRNA^Phe^ [45].

On the basis of the similarity of the structures and sequences, class I aaRSs can be further divided into three subgroups: Ia, Ib, and Ic [19,46,47] (Table 1). The enzymes IleRS, LeuRS, MetRS, ValRS, and CysRS are grouped together as class Ia, specific for the catalysis of amino acids with aliphatic side chains or containing sulfur. The members of subclass Ib comprising ArgRS, GlnRS, GluRS, and archaeal LysRS recognize polar amino acids and require the presence of corresponding tRNA for the first-step adenylate formation [20,21,22,23]. Dimeric TyrRS and TrpRS, belonging to subclass Ic, are responsible for the aminoacylation of aromatic amino acids.

The class II aaRSs can also be divided into three subgroups based on sequence identity. Subgroup IIa including HisRS, ProRS, SerRS, ThrRS, and archaeal/eukaryotic GlyRS have a homologous C-terminal anti-codon binding domain responsible for recognizing the anticodon loop of cognate tRNAs except SerRS. This latter aaRS recognizes six variant tRNAs for coding serine through the long variable arm [48]. AspRS, AsnRS, and LysRS are usually grouped together as class IIb and contain an N-terminal extension, which interacts with the anticodon stem of the corresponding tRNA in which all have a conserved uracil in the middle of the anticodon sequence. The subclass IIc consists of AlaRS, bacterial GlyRS, PheRS, SepRS, and PylRS. Most of the synthetases in this subclass are tetramers assembling as α_4_ or α_2_β_2_, with the exception of PylRS, which only forms a dimer [40,49].

## 4. Quality Control for Correct Loading of aaRSs

To ensure the fidelity of the aminoacylation reaction, aaRSs possess the ability to discriminate the specific amino acids and their cognate tRNAs in the complex intracellular environment, employing both passive substrate selectivity and active editing mechanisms [50]. Defects in aaRS proof-reading result in a series of amino acid related toxicities causing cell death in microorganisms and some neurological diseases in mammals [51,52].

Recognition of the correct tRNA by aaRSs is supported by the presence of positive identity elements, which facilitate productive interactions between enzyme and cognate tRNA, and negative elements that avoid mischarging of noncognate tRNA. While most determinants of tRNA are located in the acceptor stem and the anticodon loop, there are some determinants that are unique to specific tRNA species. These include G^-1^ recognition in tRNA^His^ [53], the deviating Levitt base pair G15:G48 in tRNA^Cys^ [54], and the wobble base pair G3:U70 in tRNA^Ala^ [55]. In the case of SerRS and LeuRS, both having six variant tRNA substrates, instead of the anticodon loop, the variable arm of the tRNA is the major determinant [50].

In comparison with the feasible discrimination of tRNA, binding of the correct amino acid is much more challenging. AaRSs achieve amino acid substrate specificity, ensuring fidelity by employing a double-sieve model. The first filter involves the preferential binding of the correct amino acid and the second involves selective editing of any mis-charged tRNA. The active site of aaRSs acts as the first sieve, preferentially activating the cognate amino acid by combining selective and direct interactions between the aaRS and the specific amino acid, and steric occlusion, which prevents the binding of larger amino acids. Activation of structurally similar proteinogenic, non-proteinogenic, and smaller amino acids is often observed but typically at a lower catalytic rate [56].

Incorrectly activated amino acids subsequently undergo a proof-reading process either by pre-transfer or post-transfer editing in the editing site, which serves as the second sieve [57,58]. Pre-transfer editing refers to the hydrolysis of mis-activated aa-AMP to amino acid and AMP prior to transfer of the amino acid to the 3′-terminal of the tRNA [58] via a tRNA-independent or tRNA-dependent pathway. Class II SerRS, ProRS, LysRS, and class I MetRS use a tRNA-independent hydrolysis pathway occurring in their active sites. For example, *E. coli* and *Saccharomyces cerevisiae* SerRSs cannot discriminate threonine (having one more methyl group in its side chain) and cysteine (substituting hydroxyl with a thiol group) from serine. It has been shown that *S. cerevisiae* SerRS catalyzes the hydrolysis of misformed Thr-AMP significantly faster than the spontaneous hydrolysis rate of this intermediate in solution, indicating that the hydrolysis of Thr-AMP occurs in the active site of SerRS [59]. However, class I IleRS and LeuRS perform pre-transfer editing to degrade the non-cognate Val-AMP and Ile-AMP intermediate, respectively, in a tRNA-dependent fashion [60].

Post-transfer editing is usually processed in a distinct domain rather than the active site in aaRSs, where the incorrect aminoacyl-tRNA species are broken down to non-cognate amino acid and tRNA. On the basis of extensive structural and biochemical studies, there are two proposed post-transfer editing models: a direct translocation pathway for class I LeuRS, IleRS, and ValRS, and a dissociation-reassociation pathway for class II ThrRS, AlaRS, and PheRS [12,50]. All LeuRS, IleRS, and ValRS possess a connective polypeptide 1 (CP1) domain, known as the editing domain, which is connected with the main enzyme body via two β-strands. As shown by crystal structures of *E. coli* LeuRS, the misacylated 3′-end of tRNA^Leu^ can be directly translocated to the editing domain, completing the hydrolysis process, while the rest of the tRNA body preserves interactions with LeuRS through significant conformational changes of the multiple domains of the enzyme [61]. This is likely the basis for the lower rate of final product aminoacyl-tRNA release for most class I families [62]. However, for class II aaRSs, the rate limiting step of aminoacylation is the aa-AMP formation, not the aa-tRNA release. Therefore, for this family, it is proposed that incorrect aa-tRNA is rapidly dissociated from the enzyme and then rebounds to perform the post-editing step. This has been evidenced by biochemical studies of recombinant fragments of *E. coli* AlaRS, which showed that AlaRS recognizes mischarged tRNA^Ala^ involving a distinct structural domain separated from that used during aminoacylation [63].

For some tRNA synthetases, the editing domains appear to be separate proteins, also known as *trans*-editing factors, which contribute to translation fidelity by hydrolyzing incorrectly aminoacylated tRNA [50]. These *trans*-editing factors, which can be considered as a third sieve, are homologous to the editing domains in class II aaRSs and mechanistically function in the same way [50,64]. To date, several autonomous editing factors have been identified for class II ProRS, ThrRS, and AlaRS based on sequence analysis. In the case of *Haemophilus influenzae*, ProRS can mischarge alanine and cysteine, but lacks the editing activity for Cys-tRNA^Pro^. However, it was found that the hydrolysis of Cys-tRNA^Pro^ is mediated by an additional protein known as YbaK [65]. Freestanding editing paralogs have also been identified for ThrRS in crenarchaea. ThrRS-cat and ThrRS-ed are encoded by two individual genes in the chromosome. The former synthesizes Thr-tRNA^Thr^, but also produces mis-charged Ser-tRNA^Thr^ as it lacks editing activity. However, ThrRS-ed lacks aminoacylation activity, but can deacylate the mis-charged Ser-tRNA^Thr^ [66]. In addition, *trans*-editing factors termed AlaXps play an important role serving as the third sieve to prevent tRNA^Ala^ being mischarged with serine or glycine [67].

## 5. Why Are aaRSs Valuable Targets for Anti-Infective Drug Discovery?

The development of antimicrobial molecules must satisfy the basic requirement of possessing potent toxicity against the targeted microbes while showing no obvious side effects on human physiology. Since protein synthesis is essential for all living organisms, selectively inhibiting any key enzymes involved in this process has proven to be one of the most effective and exploited approaches in anti-infective chemotherapy [68]. As aaRSs are responsible for producing substrates for translation, disruption of their function interrupts the process of protein biosynthesis, leading to the mortality of a pathogen.

In the last few decades, the potential of aaRS to serve as a new class of antimicrobial targets has been explored systematically [69]. This interest can be attributed to the multiple appealing characteristics of this enzyme family as follows: (1) protein translation utilizes 20 standard amino acids each with a respective aaRS providing an expanded target repertoire; (2) divergence between prokaryotic and eukaryotic cytoplasmic tRNA synthetases suggest the possibility of achieving selective inhibitors for pathogen enzymes over the human aaRSs; (3) strong sequence and structural conservation in the catalytic sites among bacterial aaRS homologs indicates that prokaryotic-specific drugs might exhibit broad-spectrum activity; (4) a wealth of known natural and synthesized aaRSs inhibitors can be used as a starting point for further optimization; (5) an abundance of available structures of prokaryotic and eukaryotic aaRSs provide a great platform for structure-based drug design and virtual screening; and (6) the aaRSs are usually soluble, express well, are feasible to purify, and can be applied for high-throughput screening (HTS) [70]. Last but not least, (7) two antibiotics, the IleRS inhibitor mupirocin [71] and the LeuRS inhibitor tavaborole [72], have been approved by the FDA for the clinical treatment of methicillin-resistant *Staphylococcus aureus* (MRSA) and fungal-infective onychomycosis, respectively, thereby proving the successful clinical viability of aaRS inhibitors as promising antimicrobials.

## 6. Natural and Synthetic aaRSs Inhibitors and Their Inhibitory Mechanisms

All known aaRSs are multidomain proteins and thereby provide diverse possibilities for targeted inhibition. These proteins present multiple druggable pockets including those of the amino acid and ATP binding sites, the tRNA binding regions, and alternative domains or insertions such as the editing domain. Numerous natural and chemically synthesized compounds targeting different aaRSs have been described [17,69,70,73,74,75,76]. The majority of inhibitors bind to one or more pockets in the highly conserved active site, where they function as competitive inhibitors of the respective amino acid and ATP substrates, or mimic the obligate aa-AMP reaction intermediate (Figure 1a). However, some inhibitors target other regions outside the catalytic domain, either allosterically disrupting the function of the active site or binding to the different regions. The expanding library of high-resolution X-ray crystal structures of prokaryotic and eukaryotic aaRSs and their complexes with inhibitors have boosted the possibilities of antimicrobial drug discovery targeting this family of enzymes. Based on the distinct binding modes of inhibitors, we will outline the discovery and inhibitory mechanisms of some representatives below.

### 6.1. Targeting the Active Site

#### 6.1.1. Targeting the Amino Acid Binding Site

**Figure 3:**
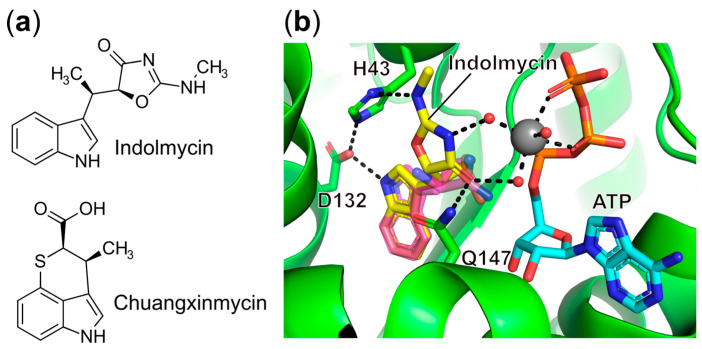
Structures of inhibitors targeting TrpRS. (**a**) Chemical structures of TrpRS inhibitors; (**b**) Superposition of the ternary structures of *B. stearothermophilus* TrpRS in complex with indolmycin and ATP (PDB entry: 5DK4) and *B. stearothermophilus* TrpRS in complex with ATP and tryptophanamide (PDB entry: 1MAU). The backbone of the former structure is shown as a cartoon representation (green) while ligands and interacting protein side-chains are shown as sticks. For clarity, ATP and a Mg^2+^ ion (grey sphere) and coordinated water molecules (red spheres) are shown for only one structure. The C-atoms of indolmycin and tryptophanamide are colored in yellow and salmon, respectively. H-bond interactions are shown as black dashed lines.

Several amino acid analogs including some with very different structures have been identified that act as competitors for particular amino acids in the active site of the respective aaRSs. One well-characterized amino acid mimicking inhibitor is indolmycin (Figure 3a), a natural tryptophan analog produced by *Streptomyces griseus*, which exhibits excellent selective inhibitory activity against *E. coli* TrpRS and *Helicobacter pylori* TrpRS with IC_50_ values of 9.25 nM and 12.2 nM over eukaryotic bovine liver TrpRS with an IC_50_ value of 4.04 mM [77]. Recently, a kinetic study demonstrated that *B. stearothermophilus* TrpRS (Bs-TrpRS) and human cytosolic TrpRS (Hc-TrpRS) showed comparable affinity for the substrate tryptophan, but Bs-TrpRS displayed 1500-fold higher affinity for indolmycin than tryptophan [78]. Structural studies showed that in the pre-transition state, Bs-TrpRS adopts a closed conformation where the Mg^2+^ ion assists in the correct positioning of the triphosphate group of ATP, bringing the α-phosphate of ATP close enough to react with Trp [79]. Indolmycin binding in Bs-TrpRS not only induces new contacts with surrounding residues in the active site compared with tryptophan, but also further stabilizes the ATP and Mg^2+^ coordination [78] (Figure 3b). In contrast, in the pre-transition state of Hc-TrpRS, both ATP and Mg^2+^ coordination, differ greatly from Bs-TrpRS as the α-phosphate of ATP must move 5.3 Å to enable nucleophilic attack of the carboxylate oxygen of tryptophan [80]. Modeling indolmycin into Hc-TrpRS points to steric hindrance, accounting for its weak binding affinity. Despite possessing good species selectivity, problems including the lack of antibacterial activity against most common bacterial species, off-target effects on tryptophan metabolism, and the emergence of resistance have hampered its clinical application [78,81].

The sulfur-containing chuangxinmycin (Figure 3a), isolated from *Actinoplanes tsinanensis*, is another tryptophan analog. In an in vivo mouse model, this compound exhibited potent activity against both Gram-negative *E. coli* and *Shigella dysenteriae* infections [82]. Although highly constrained interactions with TrpRS only allow the synthesis of sterically smaller derivatives [82], this still opens up a starting point for drug discovery targeting TrpRS. In addition, other amino acid analogs including icofungipen (also known as PLD-118, targeting IleRS), ochratoxin A (a phenylalanine analog targeting PheRS), and SB-219383 (a tyrosine analog targeting TyrRS) have been exploited as respective aaRS inhibitors [83]. Further structural studies and chemical modifications are urgently needed to increase their specificity and bacterial permeability.

#### 6.1.2. Targeting the ATP Binding Site

**Figure 4:**
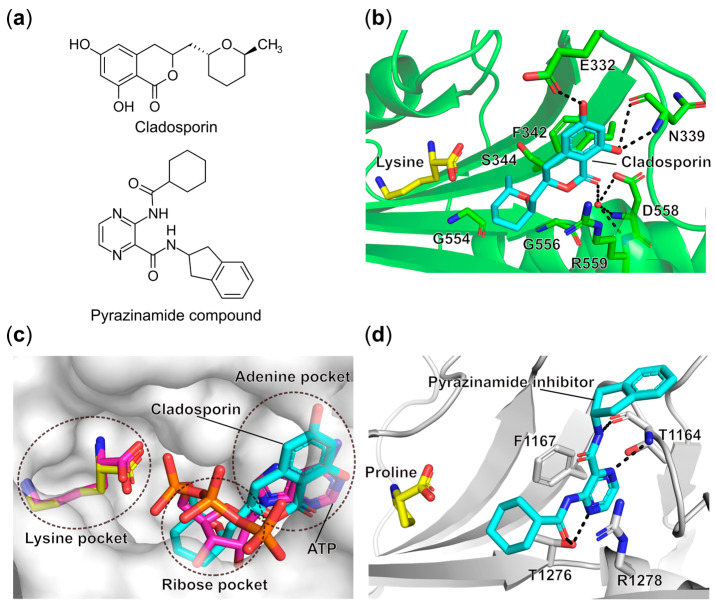
Structures of inhibitors targeting class II LysRS and ProRS. (**a**) Chemical structure of cladosporin and a pyrazinamide analog. (**b**) Structure of *P. falciparum* LysRS in complex with cladosporin and lysine (PDB entry: 4PG3). The protein backbone is shown as a green cartoon representation while ligands and interacting protein residues are shown as sticks and the water molecule is shown as a red sphere. All H-bonds are shown as black dashed lines. (**c**) Superposition of structures of *P. falciparum* LysRS in complex with cladosporin (cyan C-atoms) and lysine (yellow C-atoms) and human LysRS in complex with ATP and lysine (magenta C-atoms, PDB entry: 3BJU); Cladosporin occupies the binding sites of adenine and the ribose moiety of ATP, but has no effect on lysine substrate binding. The triphosphate group of ATP in the human complex extends toward the solvent. (**d**) Ternary structure of human ProRS in complex with the pyrazinamide analog and proline (PDB entry: 5VAD). The protein backbone is shown as a cartoon representation while the pyrazinamide inhibitor, proline, and important protein residues are shown as sticks. H-bond interactions are shown as black dashed lines.

ATP-mimetic cladosporin (Figure 4a) is a secondary metabolite isolated from several fungal genera such as *Aspergillus*, *Cladosporium*, and others. Cladosporin has been shown to have broad antimicrobial and insecticidal bioactivities [84]. The natural compound was found to strongly inhibit both blood and liver stages of the *P. falciparum* parasite without obvious toxicity on human cells and it was further proven to be >100-fold more specific in targeting *P. falciparum* cytosolic LysRS (Pf-LysRS) compared to the human homolog [85]. Crystal structures of both apo and inhibitor-bound states of Pf-LysRS showed that cladosporin occupies the adenosine binding site of the enzyme, which is competitive with the binding of ATP substrate [86,87]. The isocoumarin moiety of cladosporin is stacked with the side chain of Phe342 by a π–π interaction. The 6-OH and 8-OH groups form H-bonds with the side chain of Glu332 and backbone of Asn339, mimicking the interactions mediated by the N1 and N6 of the adenine of ATP (Figure 4b,c). In parallel, the 1-ketone group of the inhibitor establishes similar contacts with Asp558 and Arg559 via water bridges as seen for the N3 of adenine (Figure 4b) [86]. In the previous studies, two key residues Val328 and Ser344 were proposed to determine the selectivity against Pf-LysRS versus the eukaryotic yeast version in which they are substituted with Gln324 and Thr340, respectively. However, mutagenesis of these latter two residues only partially rescued the sensitivity. The ternary structure of human LysRS complexed with lysine and cladosporin proved to be indistinguishable to the equivalent complex in *P. falciparum* LysRS. Thermal shift assays revealed that in the case of Pf-LysRS, the binding affinity of cladosporin increased 625-fold in the presence of lysine, which was not observed in the human enzyme. Indeed, co-binding of lysine and cladosporin resulted in a more stable and ordered structure with relatively lower crystallographic b-factors, indicating that Pf-LysRS selectivity of this inhibitor involves lysine-dependent stabilization [87]. In addition, three small residues, Ser344, Gly554, and Gly556, at the bottom of the ATP binding pocket are responsible for the interactions with the methyltetrahydropyran moiety of cladosporin and account for the specificity against Pf-LysRS rather than other class II aaRSs [87]. The detailed dissection of the inhibition mechanism of cladosporin has inspired the investigation of its derivatives [88,89].

Human ProRS is part of a dual functional glutamyl prolyl-tRNA synthetase and inhibition of its activity has shown beneficial effects for treatments of fibrosis, cancers, and autoimmune diseases [90,91]. Therefore, human cytosolic ProRS (Hc-ProRS) is suggested to be a potential therapeutic target for these diseases. This has recently led to the identification of a novel series of pyrazinamide analogs (Figure 4a), in which the best compound exhibited 0.76 nM binding affinity to Hc-ProRS in the presence of proline [90]. Both biochemical and biophysical assays revealed that this compound is a competitive binder for ATP, but not for proline. However, the presence of proline can facilitate the binding of the compound. Structural studies show that in addition to the stacking interactions between the pyrazine group of the inhibitor and Phe1167 from motif-2 and Arg1278 from motif-3, the compound also forms direct H-bonds with the side chain of Thr1276 and backbone of Thr1164 (Figure 4d). Importantly, this compound selectively inhibits ProRS rather than another class II member ThrRS. In addition, the compound displayed potent cellular protein synthesis inhibitory activity with an IC_50_ value of 900 nM [90]. Although targeting human ProRS, these novel pyrazinamide analogs expand the chemical scaffold space of aaRS inhibitors. To the best of our knowledge, these have not been tested on microbial ProRSs, but they could be used as a template for the development of specific inhibitors of ProRS homologs from infectious agents.

#### 6.1.3. Targeting the Amino Acid and 3′-End of tRNA Bi-Substrate Binding Site

**Figure 5:**
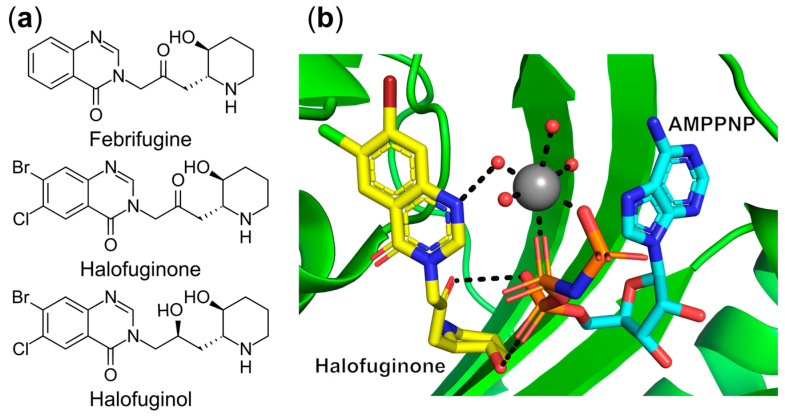
Structures of inhibitors targeting the active site of ProRS. (**a**) Chemical structures of ProRS inhibitors. (**b**) Ternary structure of *P. falciparum* ProRS in complex with halofuginone and AMPPNP (PDB entry: 4YDQ). The protein backbone is shown as a cartoon representation (green) while halofuginone (yellow C-atoms), AMPPNP (cyan C-atoms), Mg^2+^ ion (grey), and water molecules (red) are shown as sticks and spheres, respectively. H-bond interactions are shown as black dashed lines.

Febrifugine (Figure 5a), an active component isolated from Chinese herb *Dichroa febrifuga* that has been clinically used to treat malaria, showed potent in vitro inhibitory activity against both chloroquine-sensitive and chloroquine-resistant *Plasmodium falciparum* [92]. However, due to its gastrointestinal toxicity, the less toxic analog halofuginone (Figure 5a) was synthesized and was found to highly arrest *Plasmodium* parasite growth by specific interaction with and inhibition of its cytoplasmic ProRS [93]. Comparison of crystal structures of *P. falciparum* ProRS [94] in ligand-free and halofuginone-bound states demonstrated that the halofuginone occupies the binding pockets of the proline and 3′-terminal A76 nucleotide of the cognate tRNA with the assistance of ATP. The latter further stabilizes the active site conformation, facilitating the binding of halofuginone [94] (Figure 5b). This was corroborated by the 30-fold increased binding affinity of halofuginone in the presence of adenylyl-imidodiphosphate (AMPPNP), which is a non-reactive ATP analog. Nevertheless, poor selectivity of this molecule has excluded its clinical development for malaria treatment. Thus, a number of halofuginone derivatives have been reported and a new lead compound (2′S,2R,3S)-halofuginol (Figure 5a) possesses 65-fold higher selectivity against *P. falciparum* ProRS over the human homolog while still preserving excellent potency (EC_50_ = 14 nM) in the in vitro *P. berghei* ANKA strain liver-stage model [95].

#### 6.1.4. Targeting the ATP and 3′-End of tRNA Bi-Substrate Binding Site

**Figure 6:**
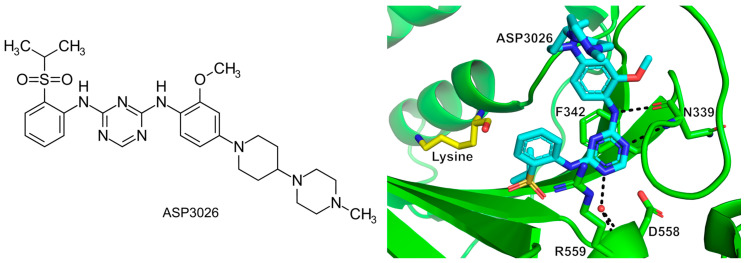
Structure of ASP3026 targeting the active site of Pf-LysRS. Left: Chemical structure of ASP3026; Right: Ternary structure of *P. falciparum* LysRS in complex with ASP3026 and lysine (PDB entry: 7BT5). The protein backbone is shown as a cartoon representation (green) while ASP3026 (cyan C-atoms), lysine (yellow C-atoms), crucial protein residues for binding, and a structured water molecule (red) are shown as sticks and a sphere, respectively. H-bond interactions are shown as black dashed lines.

To identify a new scaffold for *P. falciparum* cytoplasmic LysRS (Pf-LysRS) inhibitors, Zhou et al. screened 1215 bioactive agents from Selleck Chemicals using a high-throughput thermal shift assay [96]. Eighteen compounds were identified providing an increase in melting temperature (T_m_) above 4 °C. Among them, ASP3026 (Figure 6), a known anaplastic lymphoma kinase inhibitor that has already been evaluated for the treatment of B-cell lymphoma and solid tumors in clinical trials, was shown to be a potent novel Pf-LysRS inhibitor [96]. An ATP hydrolysis assay showed that this compound suppresses Pf-LysRS enzymatic activity with an IC_50_ value of 657 nM, which is over 380-fold more selective than for the human ortholog (IC_50_ > 250 µM). In addition, the compound inhibits blood-stage *P. falciparum* strain 3D7 growth with an IC_50_ of 5.61 μM. Further detailed analysis demonstrated that ASP3026 competes for binding with ATP and potentially prevents access of the 3′-end of tRNA to the active site. However, it has no synergistic activity in the presence of lysine nor does it interfere with the binding of the lysine substrate. The crystal structure shows that this compound occupies the ATP binding pocket with the triazine ring stacking with the class II conserved Phe342, the sulfonyl group located in the normal ribose site of ATP, and the methylpiperazine extending out of the pocket [96] (Figure 6). The latter can potentially sterically clash with the 3′-terminus of tRNA according to modeling. Given that this product has already been proven to be non-toxic and has good bioavailability as shown in clinical trials, albeit for a different purpose, this compound provides a strong edge for further speedy development as a new antimalarial. This strategy likewise opens up a new window to search for novel scaffold aaRS inhibitors by screening available ATP-competitive kinase inhibitors because they share the same ATP substrate.

#### 6.1.5. Targeting Both the Amino Acid and an Auxiliary Site

**Figure 7:**
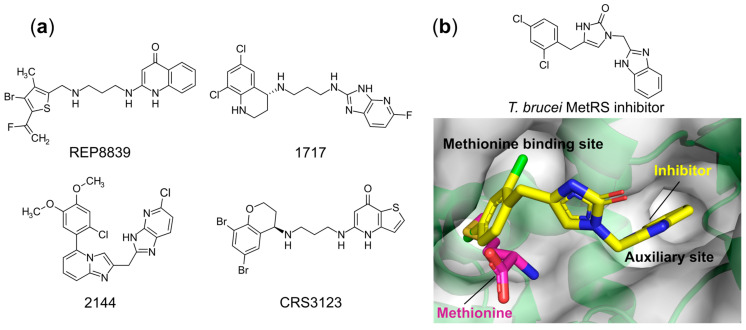
Structures of inhibitors targeting the active site of MetRS. (**a**) Chemical structures of MetRS inhibitors. (**b**) Superposition of crystal structures of *T. brucei* MetRS in complex with the inhibitor (yellow C-atoms, PDB entry: 6MES) and *T. brucei* MetRS in complex with methionine (magenta C-atoms, for clarity the protein is not depicted). The backbone structure of *T. brucei* MetRS is shown as a green cartoon representation surrounded with a transparent surface. Ligands are shown as sticks.

GlaxoSmithKline (GSK) described a series of bacterial selective aminoquinolone derivatives specifically targeting MetRS [97]. Continuing with this work, a known MetRS inhibitor REP8839 (Figure 7a), developed by Replidyne, was shown to possess promising antimicrobial activity against vancomycin-resistant *E. faecalis*, *S. aureus*, and *Streptococcus pyogenes* with MIC_90_ values of 0.25, 0.5, and 0.12 μg/mL, respectively [98,99]. This compound competes with methionine for binding in the hydrophobic amino acid pocket of MetRS, but not the ATP binding site [100]. Despite possessing excellent antibiotic potency, the poor oral bioavailability of REP8839 has restricted its application to the topical treatment of skin infections. Massive efforts have been made by Buckner [101] et al. to further optimize this scaffold to improve its oral bioavailability, pharmacokinetic properties, antibacterial potency, and selectivity versus the human ortholog, thus making better candidates for antibiotic development. The best compounds 1717 and 2144 (Figure 7a) were shown to have broad-spectrum antibacterial activities against Gram-positive pathogens *Staphylococcus*, *Enterococcus*, and *Streptococcus* strains and to be highly active against the *S. aureus* strain ATCC 29213 with MICs of 0.16 and 0.01 μg/mL, respectively. In addition, these MetRS inhibitors also demonstrated both bacteriostatic and bactericidal properties against *S. aureus* in vivo without apparent toxicity when dosed in mice for up to 10 days. Crestone developed MetRS inhibitor CRS3123 (Figure 7a) as an antibiotic for the oral treatment of Gram-positive *Clostridium difficile* infections. This compound exhibits high specificity with *C. difficile* MetRS with a K_i_ value of 0.02 nM, which is more than 1000-fold selective over the human ortholog. Recently, two phase I clinical trials of CRS3123 were completed [102,103] (clinical trial identifiers NCT01551004 and NCT02106338).

In addition to the Gram-positive bacteria, MetRS has also been validated as a druggable target in *Trypanosoma brucei* and *Leishmania donovani*, which are the causative agents of human African trypanosomiasis and leishmaniasis, respectively. All reported compounds share the similar chemical scaffold: two substituted aromatic rings connected by variant linkers [104,105,106,107,108]. However, all these compounds bind in the well-characterized sites with the same mode of action. As shown in Figure 7b, the left side of the compound occupies the enlarged methionine binding pocket while the right substituted aromatic ring is extended in an adjacent auxiliary cavity [107,108,109], which are present in the ligand-free state of all kinetoplastid MetRSs.

Based on sequence conservation and susceptibility to the inhibitors, MetRSs are classified into two distinct forms: MetRS1 (mainly found in Gram-positive bacteria and eukaryotic mitochondria) and MetRS2 (mainly found in Gram-negative bacteria, the eukaryotic cytoplasm and archaea). The aminoquinolones discussed above selectively target the MetRS1 form, but are ineffective against the other MetRS2 type enzymes. Compared to the natural substrate-bound structures, binding of the inhibitors causes large conformational changes, alongside an observed enlargement of the methionine binding pocket and the appearance of an additional “auxiliary pocket” (AP) occupied by the benzimidazole group (Figure 7b). The latter pocket is found present in the ligand-free state of a number of MetRS1 apo structures, but not in MetRS2 representatives, suggesting that the presence/absence of the AP gives rise to the observed inhibitor selectivity, where the compounds act as conformational state selectors [109]. While these compounds are effective, the existence of the aminoquinolone-resistant MetRS2 could undermine their clinical application as any targeted bacteria may acquire this isoform via horizontal gene transfer.

#### 6.1.6. Targeting the Aminoacyl-Adenylate Bi-Substrate Binding Site

**Figure 8:**
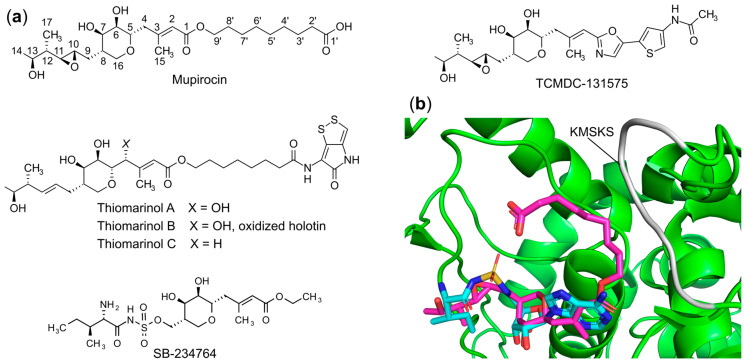
IleRS bi-substrate inhibitors. (**a**) Chemical structures of inhibitors targeting IleRS. (**b**) Comparison of the binding modes of mupirocin (magenta C-atoms, PDB entry: 1JZS) with isoleucyl-sulfamoyl adenosine (ISA, cyan C-atoms) in *T. thermophilus* IleRS. The backbone structure of IleRS is shown as a green cartoon representation with the KMSKS loop colored in grey.

In addition to the aforementioned inhibitors targeting the single substrate or amino acid/ATP and tRNA bi-substrate binding site, most of the natural and synthetic aaRS inhibitors act as non-cleavable mimics of the aminoacyl-adenylate (aa-AMP) intermediate or bind to the aa-AMP bi-substrate pocket to repress aminoacylation activity of the corresponding aaRSs. The most prominent example is mupirocin, which actually is a mixture of pseudomonic acids of which 90% is pseudomonic acid A (Figure 8a). This natural product isolated from *Pseudomonas fluorescens* NCIBM 10586 [110] functions as a potent inhibitor of IleRS [111]. Mupirocin was the first FDA-approved antibiotic specifically targeting a tRNA synthetase and it is widely used in the clinic for topical treatment of infections caused by the bacterial pathogens including methicillin-resistant *S. aureus*, *Neisseria meningitidis*, *Neisseria gonorrhoeae*, and *Haemophilus influenzae* [112]. One of the appealing characteristics of this antibiotic is that mupirocin shows approximately 8000 times more activity against bacterial IleRS than the eukaryotic homolog [113,114]. Like other antibiotics, the clinical use of mupirocin is being hampered by the appearance of resistance. Low-level resistance [115] with MIC values against *S. aureus* varying between 8 and 256 μg/mL compared with the sensitive state (MICs = 0.06 to 0.12 μg/mL) is mainly caused by single site mutations in the IleRS enzyme, while high-level resistance with MIC values above 512 μg/mL is the result of the expression of an additional IleRS2 that is found in mupirocin-producing strains [116].

The co-crystal structure of *T. thermophilus* IleRS complexed with mupirocin [71] showed that mupirocin occupies the aminoacylation pocket of the enzyme by replicating the binding of natural isoleucyl-adenylate (Ile-AMP) [71] (Figure 8b). The C12–C14 and C17 methyl group of the inhibitor are positioned in the isoleucine-specific binding site, which is lined with hydrophobic residues, via van der Waals interactions [71]. The tetrahydropyran ring (including two hydroxyl groups attached to C6 and C7, respectively) and C1–C3 moiety bind analogously to the ribose and adenine of Ile-AMP, being essential for inhibition activity (Figure 8b). The rest of the 9-hydroxynonanoic acid ester terminus of the mupirocin is sitting along the KMSKS class I signature motif and stabilizes the open conformation of this catalytic loop. Sequence alignment showed that although most residues interacting with mupirocin and Ile-AMP are the same between eubacterial, archaeal, and eukaryotic IleRSs, the two main residues His581 and Leu583 directly interacting with mupirocin are not conserved. Further mutation of these residues to the corresponding substituents found in eukaryotic IleRS or *S. aureus* type-II IleRS decreases affinity for mupirocin 10-fold, confirming their importance in selectivity determination [71].

Due to mupirocin’s strong potency and selectivity against pathogens and lack of bioavailability and stability, several analogs like thiomarinol [117,118] and TCMDC-131575 [119] (Figure 8a) have been identified serving as new scaffolds for IleRS inhibitor development. In comparison with mupirocin, thiomarinol demonstrated stronger and broader-spectrum inhibitory activity against both Gram-positive and Gram-negative pathogens, while TCMDC-131575 has been shown to target *P. falciparum* cytoplasmic IleRS.

In addition, since the epoxide-containing side group does not fully mimic all the possible interactions mediated by isoleucine and the phosphate group of the intermediate, substitutions of this arm of the compound were explored [120]. A potent femtomolar inhibitor SB-234764 (Figure 8a) was identified by replacing the epoxide-containing moiety with the isoleucyl-sulfamate group, demonstrating two-orders of magnitude greater affinity than the parent compound [120]. Unfortunately, no antibacterial activity was revealed, strongly suggesting a lack of uptake.

##### Trojan Horse aaRS Inhibitors

**Figure 9:**
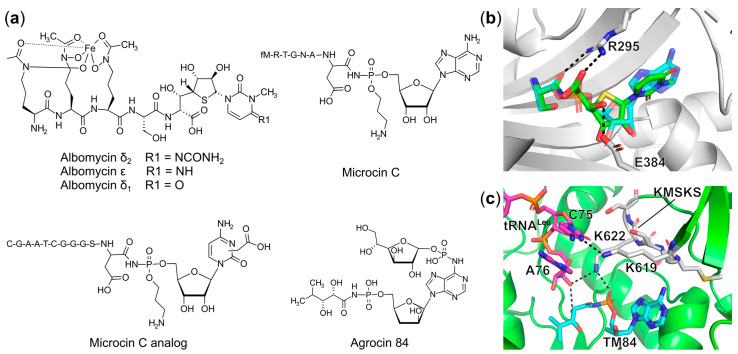
Trojan horse aaRS inhibitors. (**a**) Chemical structures of trojan horse aaRS bi-substrate inhibitors. (**b**) Comparison of the binding modes of the active component of albomycin δ1 (green C-atoms) and seryl-adenylate (Ser-AMP, cyan C-atoms) in the active site of *T. brucei* SerRS. The protein structure is shown as a grey cartoon representation with selected side chains as sticks. H-bonds are shown as black dashed lines. (**c**) Ternary structure of *E. coli* LeuRS in complex with tRNA^Leu^ (magenta C-atoms) and TM84 (cyan C-atoms; PDB entry: 3ZGZ). The protein backbone is shown as a green cartoon diagram. The KMSKS sequence motif, the inhibitor TM84, and 3′-end of tRNA^Leu^ are shown as sticks and all H-bond interactions are shown as black dashed lines.

Trojan Horse aaRS inhibitors are an important family of antibiotics containing an uptake entity that can be specifically recognized by the bacteria via their own transport systems to gain access to the cell. Once entering the cell, this moiety is readily cleaved off by the host proteolytic processes and liberates the active nucleotide toxin (also known as the “warhead”), which is usually a non-hydrolyzable analog of the aa-AMP intermediate targeting a particular tRNA synthetase, as seen in albomycin, microcin C, and agrocin 84 (Figure 9a) [70]. These fascinating Trojan Horse aaRS inhibitors are of particular interest for further development due to their inherent capability to address the poor uptake problem encountered by many other high affinity aa-AMP analogs.

Albomycin, originally isolated from *Actinomyces subtropicus*, was used in the former Soviet Union for the treatment of Gram-positive bacterial infections caused by pathogenic cocci without apparent toxicity [121]. The broader clinical application of this antibiotic was abandoned due to the rapid appearance of resistance and low production yields. Recently, it was demonstrated that, in an in vivo mouse model, albomycin effectively reduced both Gram-negative *Yersinia enterocolitica* loading in spleen and cleared Gram-positive *Streptococcus pneumoniae* in the blood [122]. Such broad-spectrum activity has thus reignited interest in this molecule. Structurally, albomycin is categorized as a peptidyl nucleoside antibiotic (Figure 9a). Several variants of albomycin including δ_1_, δ_2_, and ε, have been isolated. The N-terminus consists of a ferrichrome siderophore that is specifically taken up by bacteria via an ABC-type transport system as a mechanism to obtain iron from the environment. Once inside the cell, the active cytosine-containing component at the C-terminus is proteolytically released (Figure 9a). This non-hydrolyzable entity named as SB-217452 (corresponding to the active moiety of albomycin δ_2_) was shown to inhibit *S. aureus* SerRS with an IC_50_ value in the range of 8 nM [123] by mimicking the Ser-AMP reaction intermediate. Despite its effectivity, attempts to assess the role of different modifications of albomycin on uptake and inhibitory activity, and exploiting this knowledge to create analogous inhibitors, have been severely hampered by the inability to isolate it in suitable quantities.

To overcome large-scale production problems, and to provide a better understanding of the biochemical pathway that yields albomycin, the responsible operon in *Streptomyces* sp. ATCC 700974 was identified [124]. The current model predicts that 12 of the total 18 genes in the cluster are required for synthesis of the compound. A single gene, *abmQ*, encoding a non-ribosomal peptide synthetase, likely produces the ferrichrome siderophore. The remaining genes are predicted to be involved in the thioxylofuranosyl cytidine component. Of these only two, *abmI* and *abmE* encoding a putative N-methyltransferase and carbamoyltransferase, respectively, have their roles fully determined and are required for modification of the cytidine moiety. In 2018, the total synthesis of albomycin alongside the characterization of its antibacterial activity was reported by Lin, Z. et al. [125]. An in vitro antibacterial assessment of the synthetic compound albomycin δ_2_ demonstrated the MIC values of 3.9 ng/mL and 62.5 ng/mL against clinical pathogens like Gram-positive *S. pneumoniae* and *S. aureus* including multiple MRSA strains, proving it to be an interesting drug candidate [125]. These initial findings provide an excellent framework to develop semi-synthesis or total synthesis strategies to produce albomycin-like compounds with an expanded inhibition repertoire. Our own group accomplished total synthesis of the albomycin δ_1_ warhead and functionalized it not only with serine, but also with isoleucine and aspartic acid targeting SerRS, IleRS, and AspRS, respectively [126]. However, only the *E. coli* class II SerRS and AspRS were strongly inhibited with IC_50_ values of approximately 100 nM. No obvious effect on class I IleRS activity was observed even at 10 μM. Structural analysis showed the 5′*S*, 6′*R*-configuration of the albomycin warhead is required for binding in the active site of SerRS by maintaining the crucial interactions with the invariant motif-2 arginine and glutamate as seen for the phosphoanhydride moiety in Ser-AMP (Figure 9b).

Another well characterized Trojan Horse antibiotic is microcin C (McC), a natural product from *Enterobacteriaceae* [127,128], which contains a heptapeptide moiety connected with an adenosine via a phosphoramidate bond (Figure 9a). This phosphate is further decorated by adding a 3-aminopropyl group. The N-terminal peptide of McC acts as an entry component that is recognized by the YejABEF transporter located in the inner membrane of sensitive bacteria like *E. coli*, *Klebsiella*, *Shigella*, *Salmonella*, and *Proteus* [129]. After gaining access to the cell, the peptide entity is processed by peptidases A, B, N, and the aspartyl-adenylate analog is subsequently released as a potent AspRS inhibitor [130]. It competes with aspartic acid and ATP binding for the active site of AspRS while the 3-aminopropyl moiety is positioned to an adjacent cavity to the aspartyl site [130]. Due to its efficiency, several derivatives comprising the first six amino acids of the McC peptide linked to aminoacyl-sulfamoyl adenylates targeting AspRS, GluRS, and LeuRS were successfully synthesized. These compounds were taken up and processed by the cell, showing micromolar antibacterial activity [131,132]. Interestingly, in the McC biosynthetic gene cluster, two genes were found encoding for MccE and MccF, which catalyze acetylation of the α-amine group of the aspartyl moiety of the core inhibitor or hydrolyze the phosphoramidate bond between aspartyl and AMP in McC, respectively. These enzymes inactivate McC as a self-immunity mechanism in McC-producing strains [133].

Recently, a new McC analog, containing a carboxymethyl-cytidine (Figure 9a) instead of adenosine, was identified and isolated from *Bacillus amyloliquefaciens* DSM 7 and *Yersinia pseudotuberculosis* IP 32953 [134,135]. Although no biological activity for this compound was revealed, a chimeric compound MccA^Eco^-cxCMP was generated by fusing the carboxymethyl modified aspartyl-cytidine (cxCMP) to the *E. coli* MccA uptake peptide. This hybrid molecule demonstrated a MIC value of 20 μM against *E. coli* as determined by a spot bioactivity test. It has been proven that the presence of this carboxymethyl group on the cytosine ring in this McC analog enhances its toxicity and escapes resistance caused by acetylation of the α-amino group [134].

Agrocin 84, another example of aaRS Trojan Horse antibiotics, is secreted by the biocontrol agent *Agrobacterium radiobacter* strain K84 used to prevent the plant disease crown gall caused by the pathogen *Agrobacterium tumefaciens* [136,137]. This antibiotic consists of a stable leucyl-adenylate analog linked to a D-glucosyloxyphosphoryl entity at the N6 amine group of adenine base via a phosphoramidate bond (Figure 9a). The D-glucofuranosyloxy phosphoryl moiety mimics a plant tumor-derived substrate agrocinopine, facilitating uptake of agrocin 84 into susceptible pathogens through the opine transporter [138,139]. Once inside, agrocin 84 is processed and releases the active component named TM84, inhibiting the aminoacylation function of *A. tumefaciens* LeuRS. Of interest, recent biophysical and biochemical studies showed that in the absence of tRNA^Leu^, TM84 is a comparably weak binder to LeuRS with the K_d_ value of 152 nM; while in the presence of tRNA^Leu^, its binding affinity increases 200-fold with the K_d_ value of 0.81 nM indicating that this antibiotic possesses a unique tRNA-dependent inhibition mechanism [140]. The ternary complex of *E. coli* LeuRS, TM84 and tRNA^Leu^ further revealed that the active toxin TM84 traps the LeuRS-tRNA^Leu^ complex in a novel aminoacylation-like closed conformation [140]. Comparison with the ternary structure of *E. coli* LeuRS in complex with tRNA^Leu^ and leucyl-sulfamoyl adenylate (LSA) captured in the aminoacylation conformation showed only subtle differences. However, three unique interactions with the class I conserved KMSKS signature motif and 3′-end of tRNA^Leu^ seen for the TM84-bound structure can be noted including H-bonds mediated by Lys622 to the phosphate of TM84, the C3′-OH of terminal A73 to the sidechain C2-OH of TM84, and Lys619 to the penultimate C75 of tRNA^Leu^, stabilizing the overall aminoacylation-like conformation (Figure 9c). This in turn supported the tRNA^Leu^-assisted TM84 binding mechanism.

To avoid cell suicide, an additional non-essential LeuRS (referred to AgnB2) is encoded in the TM84-producing *A. radiobacter*. A similar phenomenon has been reported for other natural aaRSs inhibitors such as albomycin [141], borrelidin [142], mupirocin [143], and indolmycin [144]. Biochemical and biophysical studies revealed that AgnB2 achieves resistance to TM84 not only by decreasing its binding affinity for both tRNA^Leu^ and the inhibitor alone, but also reduces the effect of tRNA^Leu^ on the inhibitor’s binding for the enzyme without affecting its overall aminoacylation catalytic activity [145]. Structural comparison of the TM84 resistant AgnB2 and TM84 sensitive *E. coli* LeuRS in combination with mutagenesis studies revealed the key role of some residues in the determination of AgnB2 resistance to this antibiotic [145]. Taken together, the high potency and detailed structural studies of these Trojan Horse aaRS inhibitors are a solid basis for further development of these as lead compounds.

##### Synthetic Bi-Substrate aaRS Inhibitors

**Figure 10:**
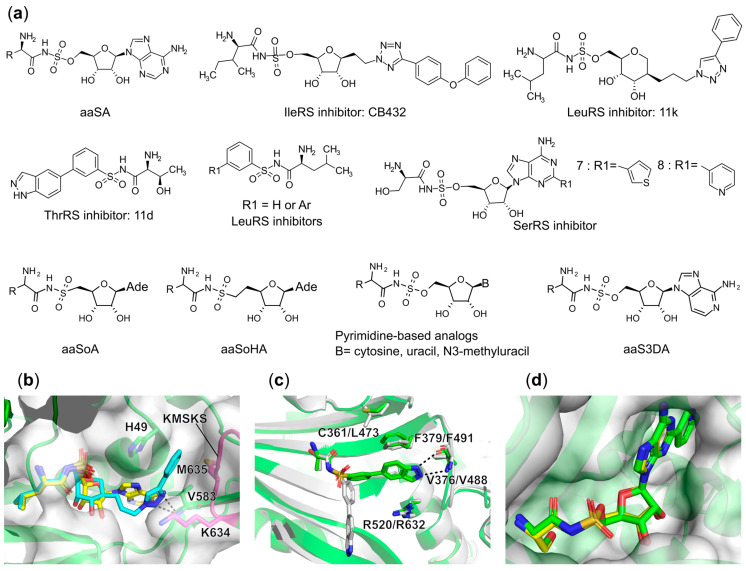
Synthetic aaRS bi-substrate inhibitors. (**a**) Chemical structures of synthetic aaRS inhibitors. The abbreviations are defined as follows: aaSA (aminoacyl-sulfamoyl adenosine), aaSoA (5′-(N-aminoacyl)-sulfonamido-5′-deoxyadenosine), aaSoHA (N-L-aminoacyl-C-5′-adenosyl-methansulfonamide), and aaS3DA (aminoacyl-sulfamoyl 3-deazaadenosine). The R group corresponds to the side chain of a proteinogenic amino acid (aa) while Ade represents the natural adenine base. (**b**) Comparison of the binding modes of compound **11k** (cyan C-atoms; PDB entry: 6YKL) and LSA (yellow C-atoms; PDB entry: 6Q89) in the aminoacylation site of *N. gonorrhoeae* LeuRS. The protein structure of the former complex is shown as a green cartoon representation covered by a transparent surface. The KMSKS loop region is colored in magenta. H-bonds are shown as black dashed lines. (**c**) Superposition of **11d** bound to *E. coli* (green, PDB entry: 4HWR) and human ThrRS (grey, PDB entry: 4HWT). Important residues at equivalent positions in *E. coli* ThrRS and human ThrRS are indicated. (**d**) Superposition of *E. coli* SerRS in complex with seryl-sulfamoyl adenosine (SSA, yellow C-atoms, PDB entry: 6R1M) and compound **8** (green C-atoms; PDB entry: 6R1O). The protein backbone of the latter complex is shown as a cartoon representation (green) covered by a transparent surface.

Aside from natural bi-substrate aaRS inhibitors, most direct analogs of aa-AMP have been created by replacing the labile phosphoanhydride linker of the obligatory reaction intermediate with a chemically stable, non-hydrolyzable ester, sulfamate, or phosphonate linkage [146]. The most thoroughly investigated and potent analogs are those bearing a 5′-O-sulfamate moiety, referring to aminoacyl-sulfamoyl adenylate (aaSA, Figure 10a). These compounds have proven to be tight binders to the corresponding aaRS with low nanomolar affinity, retaining all the interactions observed for aa-AMP in crystal structures [70]. However, the lack of inhibitory selectivity between pathogenic and host enzymes and limitation of whole-cell antibacterial activity due to poor permeability have ruled out their further application for clinical use. To obtain better selectivity and in vivo activity, numerous modifications have been investigated based on this aaSA scaffold.

One successful synthetic intermediate analog example is the IleRS inhibitor CB432 produced by Cubist Pharmaceuticals (Figure 10a). Substitution of the adenine base with a phenyltetrazole moiety yielded a molecule that demonstrated a 60- to 1100-fold selectivity against pathogenic bacterial IleRS relative to the human ortholog in vitro [74]. This compound also displayed excellent antibacterial activity with MIC values of 0.5, 10, and 10 μg/mL for *S. pyogenes*, *S. aureus*, and *E. coli*, respectively [147]. Increased MIC values for *S. aureus* cell growth were detected with increasing concentrations of isoleucine substrate in the culture, which proved that the intracellular target of CB432 was IleRS. Despite their efficiency, clinical investigation of this candidate was discontinued because of poor bioavailability, which in part was reported to be due to its binding to serum albumin [74].

Based on this research, De Ruysscher et al. recently described a series of anhydrohexitol-based LeuRS inhibitors carrying substituted triazoles, in which the phenyl-substituted compound **11k** (Figure 10a) displayed the best inhibitory properties with a K_i_^app^ of 2.5 nM against *E. coli* LeuRS [148]. X-ray crystallographic studies of *N. gonorrhoeae* LeuRS in complex with several of the inhibitors further highlighted the crucial interactions for this series of compounds. Briefly, this class of inhibitors bind in the aminoacylation site of LeuRS where the leucyl-anhydrohexitol fully mimics the interactions observed for leucyl-sulfamoyl in LSA while the modified triazole binds in the cavity generated between the HIGH and KMSKS sequence motifs, with the latter KMSKS loop in an open conformation (Figure 10b). This is reminiscent of the binding mode of mupirocin with IleRS (Figure 8b) [71].

More recently, studies exploring the structure–activity relationships (SAR) of benzenesulfonamide based inhibitors that bind both the amino acid and ATP pockets of ThrRS [149] and LeuRS [150] have been described. In the case of the ThrRS specific compounds developed by Trius Therapeutics, some displayed excellent selectivity against bacterial ThrRS compared to the human ortholog. Among them, the most promising candidate, compound **11d** (Figure 10a) demonstrated more than 270-fold selectivity against ThrRS in *E. coli*, *Burkholderia thailandensis*, and *Yersinia pestis* with K_i_ values in the range of 132–182 nM versus a K_i_ above 50 μM for the human enzyme. A co-crystal structure of *E. coli* ThrRS in complex with **11d** showed that it adopts the same conformation and induces the typical conformational changes of protein residues in the active site as seen for the intermediate analog threonyl-sulfamoyl adenosine (TSA) (Figure 10c). However, in the human ortholog, **11d** binds in an alternative conformation in which the threonyl moiety remains in the threonine binding pocket while the indazole group is pointing away from the ATP binding site (Figure 10c). Superposition of these two structures showed that the conformation and lack of flexibility of Phe491 in human ThrRS, a highly conserved residue in class II aaRSs responsible for stacking with the adenine base of ATP, is immobilized by Leu473 and thus clashes with the compound indazole group. It further results in the loss of interaction with the backbone of Val488, forcing **11d** to adopt a much less energetically favorable binding state in the human enzyme [149] (Figure 10c). Moderate antibacterial activity of these compounds has been observed against wild type *H. influenzae* and efflux-deficient mutants of *E. coli* and *B. thailandensis* [149].

Scientists at Oxford Drug Design reported compounds (Figure 10a) similar to the Trius **11d** that target bacterial LeuRS. Most of them show tight binding affinity (<100 nM) to *E. coli* LeuRS as measured by isothermal titration calorimetry (ITC) [150]. Interestingly, enzymatic assays demonstrated that the best compound, N-leucinyl benzenesulfonamide (Figure 10a, R1 = H), inhibited *E. coli* LeuRS (IC_50_ = 35 nM) with 150- and 30-fold selectivity over the orthologous enzymes from the Gram-positive organism *S. aureus* and the human cytosolic LeuRSs, respectively. Furthermore, in a whole cell assay, this compound also exhibited good antibacterial activity against the *E. coli* ATCC 25922 strain with a MIC value of 8 μg/mL [150].

Employing structure-based drug design (SBDD) and bioinformatics approaches, a novel series of selective bacterial SerRS inhibitors were synthesized [151] (Figure 10a). Superposing the structures of SSA bound to SerRS from *E. coli*, *S. aureus*, and human cytoplasm showed a high degree of similarity, but a small extension in the hydrophobic cavity adjacent to the C2 position of SSA was only present in prokaryotic SerRS and not in human enzymes. Bioinformatic analysis revealed that the boundary between the canonical catalytic site and this extended pocket is defined by a glycine residue (one residue upstream of the highly conserved class II arginine in motif-3 that is responsible for stacking interaction with the adenine base of ATP) instead of an amino acid with a side chain like threonine in the human homolog. Therefore, the newly designed compounds focus on modification at the C2 position of adenine, based on the SSA chemical framework [151] (Figure 10a). Enzymatic assay results demonstrated that any changes at the C2 position decreased the binding affinity with respect to SSA, but hydrophobic substituents significantly enhanced selectivity for the bacterial enzyme over human SerRS. Although the best compounds **7** and **8** (Figure 10a) displayed only a range of 1.24–6.65 μM of inhibitory activity for *E. coli* and *S. aureus* SerRSs, they were still more than two orders of magnitude selective versus the human homolog. The co-crystal structure of compound **8** bound to *E. coli* SerRS clearly shows that the compound binds in the same mode as SSA and the 3-pyridyl moiety is extended in the hydrophobic pocket, which is likely to sterically clash with Thr434 in the human SerRS. Unfortunately, this series of compounds showed no obvious antibacterial activity, likely resulting from their poor permeability. This can probably be overcome by applying a Trojan Horse antibiotic strategy or further lead modification.

In our own work, several modifications based on the aaSA scaffold have been evaluated for their improvement in stability and inhibitory activity. The 5′-oxygen in the sulfamoyl linker was deleted resulting in a series of aaSoA analogs [152] (Figure 10a). This decreased the electrophilicity of the 5′-carbon atom as the resulting sulfonamide moiety is a poorer leaving group in comparison with the original sulfamoyl. Unfortunately, the majority of these aaSoA congeners are not able to efficiently inhibit their target aaRS. Subsequently, we substituted 5′-oxygen with a carbon atom to generate two similar isosteres [153] (aaSoHA, Figure 10a) targeting class I IleRS and class II SerRS, respectively. Evaluation of their in vitro enzyme inhibitory activity showed that the isoleucyl-sulfonamide analog retained its potency compared to the parent sulfamoyl derivative (ISA), while an almost complete loss of activity was observed for the seryl congener. Modeling of the compound in the active site of the corresponding protein structure demonstrated that this is likely a result of unfavorable eclipsed conformation around the 6′-CH_2_ group of the seryl congener, leading to a steric clash upon binding with the protein, which is absent in the case of the Class I synthetases. Additionally, adenine base modifications or substitutions have been investigated [154,155,156]. Among these, 3-deazaadosine analogs (aaS3DAs) targeting both Class I and II aaRS representatives were synthesized but showed inhibitory preference for Class I aaRSs rather than Class II members. Interestingly, substitution of the adenine base with cytosine, uracil, and N^3^-methyuracil inspired by the aforementioned natural antibiotics albomycin and the McC analog were also synthesized (Figure 10a). Despite showing decreased inhibitory activity relative to their respective aaSA congeners, most of the analogs still inhibited representatives from both aaRS classes in the lower nanomolar range. Structural studies highlighted a subtle interplay between the base moiety and the target enzyme in defining relative inhibitory activity.

For all of these synthetic aaRS inhibitors, despite some showing excellent enzyme inhibitory activity, selectivity, and antibacterial potency, the α-amine group of the aminoacyl moiety in these compounds appears to be their Achilles heel [155,157,158]. It has been reported that *E. coli* MccE and RimL, two acetyl-CoA-dependent acetyltransferases, are able to detoxify several nonhydrolyzable aminoacyl adenylates including both natural McC, albomycin, and synthetic non-hydrolyzable aminoacyl adenylates (for instance, aspartyl-sulfamoyl adenosine) in a mechanism to protect bacteria from various toxic aminoacyl nucleotides [155,157,158]. To prevent this resistance mechanism, Vondenhoff et al. synthesized N-methylated congeners for LSA and GSA (glycyl-sulfamoyl adenosine) but observed a significant reduction in inhibitory activity, showing that even a small modification of this amine is detrimental to binding [159]. However, the aforementioned carboxymethyl cytidine-containing McC analog (Figure 9a) has been shown to be resistant to MccE catalyzed N-acetylation [134]. Characterization of sulfamoyl based analogs mimicking this pyrimidine suggests that the alternative base has a slightly reduced aaRS affinity compared to the canonical purine [155,156], therefore it is likely that this base-substitution has a greater effect on binding to MccE. The effect on scaffold substitution should therefore be investigated further to identify ways of minimizing, or escaping, N-acetyltransferase catalyzed detoxification of this group of aaRS inhibitors.

#### 6.1.7. Targeting Multiple Binding Sites

**Figure 11:**
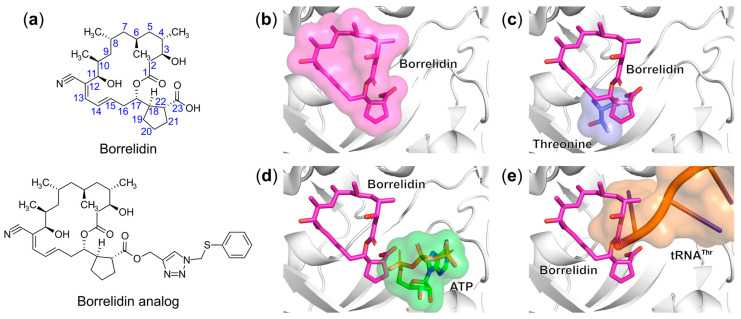
Structures of inhibitors targeting ThrRS. (**a**) Chemical structures of borrelidin and its analog. (**b**) Crystal structure of *E. coli* ThrRS in complex with borrelidin (PDB entry: 4P3P). The protein backbone is shown as a cartoon representation (grey) while borrelidin is shown as sticks (magenta C-atoms) covered by a semi-transparent molecular surface. (**c**) Threonine binding site of superimposed *E. coli* ThrRS-borrelidin with ThrRS-threonine (PDB entry: 1EVK) structures. Threonine is shown as a stick model (slate C-atoms) covered by a semi-transparent surface. (**d**) ATP binding site of superimposed *E. coli* ThrRS-borrelidin with *S. aureus* ThrRS-ATP (PDB entry: 1NYR) structures. ATP is shown as a stick representation (green C-atoms) covered by a semi-transparent molecular surface. (**e**) The tRNA^Thr^ binding site of superimposed *E. coli* ThrRS-borrelidin with ThrRS-tRNA^Thr^ (PDB entry: 1QF6) structures. The tRNA^Thr^ is shown as an orange cartoon diagram covered by a semi-transparent molecular surface.

Borrelidin, a C18 polyketide natural compound (Figure 11a) produced by *Streptomyces rochei* or *Streptomyces parvulus*, displays broad-spectrum antibacterial, antiviral, antifungal, insecticidal, and herbicidal bioactivities [160,161,162]. More recently, it has been found that borrelidin effectively inhibits parasitic growth of both the drug-sensitive FCR3 strain and drug-resistant K1 strain of *P. falciparum* in vitro with IC_50_ values of 1.8 nM and 1.9 nM, respectively [163]. Despite these strong activities, the significant toxicity of borrelidin toward human cells [164] (IC_50_ = 345 nM against human HEK293 cells in a 72 h test) is a major deterrent to its clinical application. Therefore, several SAR studies have been carried out based on attempts to attenuate its cytotoxicity while retaining its antimalarial activity. In 2013, Sugawara et al. generated a series of borrelidin derivatives by extending the size of the molecule at its carboxylic acid group (Figure 11a). The most selective compound, bearing a CH_2_SPh group connected with the carboxylic acid moiety via a triazole linker (Figure 11a), showed more than 20,000-fold specificity with IC_50_ values of 0.85 ng/mL against the FCR3 strain and 0.031 ng/mL against the K1 strain over human diploid embryonic MRC-5 cells [165]. This latter compound is a promising candidate for further development as a clinically significant antimalarial drug.

Biochemical studies have demonstrated that the main target of borrelidin is ThrRS. It inhibits the threonine activation step of bacterial and human ThrRS with a K_i_ value of about 4 nM and 7 nM in vitro, respectively [166]. Co-crystal structures of *E. coli* and human ThrRS in complex with borrelidin revealed that the bound compound can compete with the binding of threonine, ATP, and the 3′-end of cognate tRNA acceptor arm [167] (Figure 11b–e). The C18–C23 region of borrelidin overlaps with the carboxylate group of the substrate threonine while the C22–C23 and C1–C5 atoms sterically clash with the binding of α-phosphate of ATP and the 3′-terminal adenosine of tRNA^Thr^, respectively. Interestingly, borrelidin was also found to extend into a fourth pocket within the ThrRS active site, which was not observed in other structures of the same enzyme, and created upon binding of this molecule [167]. The addition of one of the three physiological substrates at higher concentrations rescued the enzymatic activity of ThrRS, indicating that borrelidin functions as a triple-competitive inhibitor. This unique and attractive inhibition mechanism of borrelidin opens up a new approach for the development of multifunctional inhibitors.

### 6.2. Targeting the Editing Site

**Figure 12:**
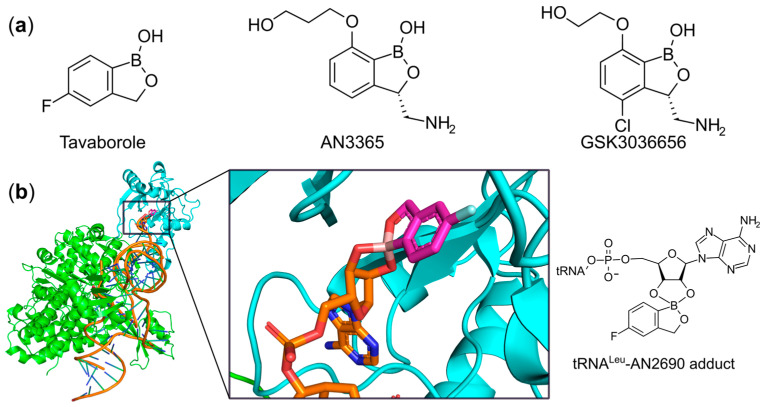
Structures of inhibitors targeting the editing domain of LeuRS. (**a**) Chemical structures of LeuRS inhibitors. (**b**) Ternary structure of *Thermus thermophilus* LeuRS in complex with tavaborole and tRNA^Leu^ (PDB entry: 2V0G). The protein backbone and tRNA^Leu^ are shown as cartoon representations with the editing domain of the former colored in cyan and the enzyme body in green. Inset: the terminal adenosine of tRNA^Leu^ (orange C-atoms) and tavaborole (magenta C-atoms) form an adduct shown as sticks, the 2D-representation of the adduct is shown on the right.

Some aaRSs such as LeuRS, ValRS, and IleRS perform a proof-reading function in an extra editing domain distal from the active site [40,50]. The aminoacylated 3′-CCA-end of cognate tRNA is preferentially translocated from the catalytic domain to the editing site where the mischarged product is hydrolyzed. The design of compounds targeting the editing site to prevent the binding of tRNA or to trap it in this domain could be an alternative approach to hamper the aminoacylation activity of aaRSs. The first successful case was AN2690 (Figure 12a), known as tavaborole, which was approved by the FDA in 2014 for the clinical treatment of the fungal disease onychomycosis [168]. Mechanistically, AN2690 specifically inhibits fungal LeuRS by forming a stable boron-mediated tRNA^Leu^-AN2690 adduct in the editing site. Therefore, it traps the 3′-terminus of the tRNA^Leu^ isoacceptor, prevents subsequent LeuRS catalytic turnover, and consequently blocks protein synthesis [72] (Figure 12b).

Due to the remarkable selectivity of this scaffold and the feasibility or easily adding chemical modifications to it, AN2690 derivatives targeting other pathogens are being actively sought. Benzoxaborole derivatives have been shown to be great potential inhibitors against *S. pneumoniae* and *T. brucei* LeuRSs [169]. AN3365 (also known as GSK2251052) was developed as treatment for Gram-negative bacterial infections [170]. However, its development was halted in phase II clinical trials due to the rapid emergence of resistance [171]. Furthermore, a series of novel 3-aminomethyl-4-halogeno-benzoxaboroles were synthesized and preclinically evaluated by GSK as selective inhibitors against *Mycobacterium tuberculosis* LeuRS (Mtb-LeuRS) [172]. The promising lead compound GSK3036656 (Figure 12a) demonstrated a MIC value of 23.5 ng/mL against *M. tuberculosis* strain H37Rv and an IC_50_ value of 58.8 ng/mL against Mtb-LeuRS in vitro. In addition, this compound showed no serious side effects following single or multiple doses and was selected for further clinical studies [172]. Most recently a series of benzhydrol-oxaborole derivatives were identified that selectively inhibited *S. pneumoniae* LeuRS and demonstrated anti-pneumococcal activity, with the best compound having a MIC of 4 μg/mL [173].

### 6.3. Targeting the tRNA or the aaRS–tRNA Interface

Another important approach to inhibit the activity of aaRSs is to disrupt the interactions between the respective aaRS and its canonical tRNA. It is worth noting that tRNA is a sophisticated macromolecule making numerous interactions with multiple domains of the enzyme. Due to the evolutionary divergences of tRNA recognition and binding modes between distant phylogenies, in addition to interfering with the 3′-end of tRNA as discussed above for tavaborole and halofuginone, direct drug binding to tRNA or the specific aaRS–tRNA interface is a viable strategy to design novel classes of species-selective antimicrobials [174]. Despite these advantages, rational drug design for targeting tRNA and/or its interaction with aaRS remains less explored.

**Figure 13:**
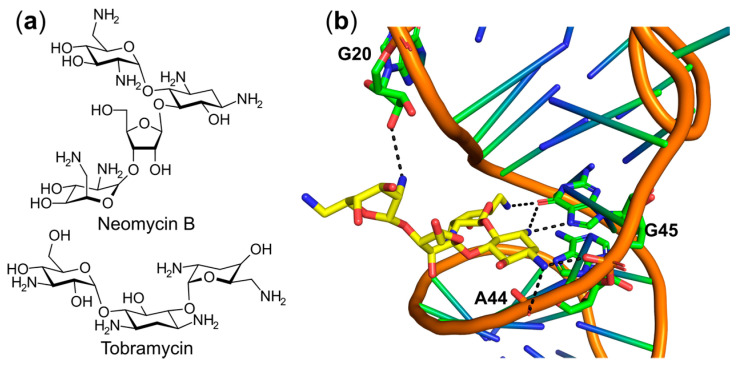
Structures of inhibitors bound to tRNA. (**a**) Chemical structures of aminoglycoside inhibitors targeting tRNA^Phe^ and tRNA^Asp^; (**b**) Structure of yeast tRNA^Phe^ bound to neomycin B (PDB entry: 1I9V). The ligand and interacting bases of tRNA^Phe^ are shown as sticks and the C-atoms colored in yellow and green, respectively. tRNA^Phe^ backbone atoms and remaining bases are shown as cartoon representations.

Aminoglycoside antibiotics neomycin B and tobramycin (Figure 13a), originally used for inhibiting protein translation by binding to the aminoacyl site of the prokaryotic 16S ribosomal RNA subunit, were shown to competitively inhibit the in vitro aminoacylation of *E. coli* tRNA^Phe^ (K_i_ ~ 300 μM) [175] and yeast tRNA^Asp^ (K_d_ = 267 nM) [176] without affecting the first-step aminoacyl-adenylate formation. Co-crystal structure of yeast tRNA^Phe^ with neomycin B demonstrated that neomycin B occupies an important divalent metal ion binding pocket in the tRNA molecule, which blocks its interaction with PheRS [175] (Figure 13b). However, binding of tobramycin leads to conformational changes in yeast tRNA^Asp^, subsequently inhibiting aminoacylation of AspRS [176].

## 7. Conclusions and Future Perspectives

Infectious diseases are an ever-growing threat to public health due to the rapid emergence and spread of antimicrobial resistance. To fend off this burgeoning problem, the identification and development of novel antibiotics targeting new drug targets is of great importance. The aaRSs play an essential role in protein synthesis and the enzyme class has been clinically validated as a suitable target for drug development. The large multi-substrate binding pockets of aaRSs are responsible for accommodating the binding of the respective amino acid, ATP, and 3′-end of cognate tRNA. Therefore, these enzymes can be inhibited via various distinct mechanisms. As outlined, numerous aaRS inhibitors have been discovered and their inhibitory mechanisms have been clearly elucidated over the last decades. Building onto these efforts, extensive structural information for different aaRS class members can provide a great platform for the structure-based drug design (SBDD) of novel potent aaRS inhibitors. In parallel, currently available aaRSs crystals can be effectively applied for fragment screening to identify new chemical scaffold hits. Additionally, the ligand-bound structures can provide a useful tool for in silico docking studies. Furthermore, the structural dynamics of these enzymes during catalysis should be explored in greater detail. It has been recognized that considerable changes in pocket size are seen in LeuRS [61] and TrpRS [80] ligand-bound structures and a new pocket, positioned away from the active site, was observed for inhibitor-bound *M. tuberculosis* MetRS [177]. A better understanding of this process could be used to expand the chemical space of inhibitors.

Aside from the above-mentioned strategies for drug design, three important aspects should also be considered to attain a suitable lead molecule: (1) improve species-selectivity to reduce toxicity; (2) enhance bacterial cell permeability to achieve in vivo activity; and (3) minimization of resistance generation, which will extend the lifespan of newly found antimicrobials.

Despite the discovery of numerous potent aaRS inhibitors, a common failure in their further development is the lack of sufficient selectivity. The human genome encodes for 36 distinct mitochondrial and cytoplasmic aaRSs. The mitochondrial aaRSs are of eubacterial origin, and therefore may be inhibited by compounds targeting bacterial enzymes. However, the mitochondrial aaRSs demonstrate slower catalytic rates than their cytosolic or bacterial homologs. For example, the aminoacylation capacity of LeuRS is approximately 250- to 400-fold lower than that detected for the corresponding aaRSs of cytoplasmic and bacterial origins [178]. This reflects lower protein translation rates in mitochondria, indicating that the inhibition of mitochondrial tRNA synthetases is likely less toxic than bacterial ones. In addition, the double-membrane system surrounding mitochondria creates an additional penetration barrier for drugs. The short-term antibiotic treatment course (generally less than 10 days) makes the accumulation of mitochondrial toxicity tolerable [179].

Ideally, the inhibition of mitochondrial enzymes should be tested and avoided in the beginning of antimicrobial development. Structural and sequence comparison between human mitochondrial and bacterial homologs is often the key to identify the important elements or exploitable differences determining the selectivity. However, as structures of only a few human aaRSs are available, additional structural studies of human tRNA synthetases are essential. In addition, during the initial development phase, compounds can be counter-screened against the purified homologs from humans. This is not always trivial, as the typically larger human enzymes can be difficult to produce in suitable quantities and may require eukaryotic tRNA for activity. To overcome these issues, Omnia Molecular Ltd. described a cell-based reporter assay, which is designed to monitor target bacterial aaRS activity inside human cells. This method is based on the use of a gene coding for a luciferase reporter whose sequence is interrupted by a stop codon. This stop codon can only be translated by an anticodon modified tRNA, which is specifically aminoacylated by the expressed bacterial enzyme. Positive hits have to cross the lipid membrane of the human cell and selectively inhibit the target aaRS while demonstrating low cytotoxicity [180].

A second common problem with the current compounds is the lack of target cell uptake. Chemical structures of the antimicrobials on the market are strikingly different when analyzing their molecular weight, lipophilicity, flexibility, or charge distribution. This is likely the result of the special characteristics of bacterial cell walls and membranes, which on the one hand, protect bacteria from toxins in the surrounding environment and, on the other hand, need to allow for the uptake of nutrients and excretion of waste. Therefore, optimal guidelines for the development of antimicrobials are required and are still not clear. In attempts to overcome this low permeability issue, several approaches have been proposed such as using carriers like nanoparticles, tuning the physiochemical properties of inhibitors [181], or using a Trojan Horse strategy by attaching a siderophore or a peptide module to the active compounds [182,183]. The application of the latter Trojan Horse strategy could also help to improve species selectivity.

Resistance to antimicrobials is a major concern and the inevitable consequence of natural selection. Eventual resistance to any novel aaRS inhibitors would not be an exception and can only be expected to be delayed. Mechanistically, the predominant modes of resistance to current compounds include the acquisition of self-immunity aaRS genes identified in natural antibiotic producing strains that normally prevent suicide [141,142,143,144,145], point mutations of the ligand binding site on the aaRS [115], and inactivation by enzymes like N-acetyltransferases [157,158]. Modification of a compound can overcome these resistance mechanisms, for example, Serebryakova et al. identified a carboxymethyl modified cytidine AspRS inhibitor that escapes the inactivation of MccE, an N-acetyltransferase [135]. Therefore, the ease of the chemical modification of a particular chemical scaffold should also be part of the considerations when selecting lead candidates. Another option to reduce the emergence of resistance would be to identify multi-synthetase inhibitors. Although members of the same aaRS class can respond differently to analogous compounds [154,155,156], one could examine target combinations such as AspRS and AsnRS or GluRS and GlnRS, which share high intermediate structure similarity, and can therefore be envisaged for inhibition by a single molecule. In addition, it has been proven that combination therapy, employing two aaRS inhibitors targeting two different aaRS enzymes, prevents the rapid emergence of spontaneous bacterial resistance [184].

In conclusion, we are confident that, combined, all these efforts provide a useful framework for the successful development of selective, potent, and in vivo active aaRS inhibitors as clinical antimicrobials.

## Figures and Tables

**Table 1:** Subgroups of class I and class II aminoacyl-tRNA synthetases (aaRSs).

Class I aaRSs			Class II aaRSs		
Ia	Ib	Ic	IIa	IIb	IIc
CysRS	ArgRS	TrpRS	GlyRS ^2^	AsnRS	AlaRS
IleRS	GlnRS	TyrRS	HisRS	AspRS	GlyRS ^4^
LeuRS	GluRS		ProRS	LysRS ^3^	PheRS
MetRS	LysRS ^1^		SerRS		PylRS
ValRS			ThrRS		SepRS

^1^ Monomeric LysRSs are found in many archaea and some bacteria. ^2^ Archaeal and eukaryotic GlyRSs are homodimers belonging to Subclass IIa. ^3^ Class II LysRSs are dimeric. ^4^ Bacterial GlyRSs are tetramers containing α_2_β_2_ subunits.

## Data Availability

Not applicable.

## Abbreviations

aa-AMP	aminoacyl-adenylate
aaRS	aminoacyl-tRNA synthetase
aa-tRNA	aminoacyl-tRNA
aaS3DA	aminoacyl sulfamoyl 3-deazaadenosine
aaSA	aminoacyl-sulfamoyl adenosine
aaSoA	5′-(N-aminoacyl)-sulfonamido-5′-deoxyadenosine
aaSoHA	N-L-aminoacyl-C-5′-adenosyl-methansulfonamide
AdT	amidotransferase
AlaRS	alanyl-tRNA synthetase
AMP	adenosine monophosphate
AMPPNP	adenylyl-imidodiphosphate
AMR	antimicrobial resistance
AP	auxiliary pocket
ArgRS	arginyl-tRNA synthetase
AsnRS	asparaginyl-tRNA synthetase
AspRS	aspartyl-tRNA synthetase
ATP	adenosine triphosphate
CP1	connective polypeptide 1
CysRS	cysteinyl-RNA synthetase
DNA	Deoxyribonucleic acid
EC_50_	half maximal effective concentration
EPRS	glutamyl prolyl-tRNA synthetase
ESBL	extended spectrum β-lactamase
FDA	Food and Drug Administration
GlnRS	glutaminyl-tRNA synthetase
GluRS	glutamyl-tRNA synthetase
GlyRS	glycyl-tRNA synthetase
GSA	glycyl-sulfamoyl adenosine
GSK	GlaxoSmithKline
HisRS	histidyl-tRNA synthetase
HTS	high-throughput screening
IC_50_	half maximal inhibitory concentration
IleRS	isoleucyl-tRNA synthetase
ISA	isoleucyl-sulfamoyl adenosine
ITC	isothermal titration calorimetry
LeuRS	leucyl-tRNA synthetase
LSA	leucyl-sulfamoyl adenosine
LysRS	lysyl-tRNA synthetase
McC	microcin C
MDR	multidrug resistance
MetRS	methionyl-tRNA synthetase
MIC	minimum inhibitory concentration
MRSA	methicillin-resistant *Staphylococcus aureus*
Mtb	*Mycobacterium tuberculosis*
PDB	protein data bank
PheRS	phenylalanyl-tRNA synthetase
PPi	pyrophosphate
ProRS	prolyl-tRNA synthetase
PylRS	pyrrolysyl-tRNA synthetase
RF	Rossmann fold
RNA	ribonucleic acid
SAR	Structure-activity relationship
SBDD	structure-based drug design
SepRS	phosphoseryl-tRNA synthetase
SerRS	seryl-tRNA synthetase
SSA	seryl-sulfamoyl adenosine
ThrRS	threonyl-tRNA synthetase
T_m_	melting temperature
tRNA	transfer RNA
TrpRS	tryptophanyl-tRNA synthetase
TSA	threonyl-sulfamoyl adenosine
TyrRS	tyrosyl-tRNA synthetase
ValRS	valyl-tRNA synthetase
WHO	World Health Organization
